# A patient-derived orthotopic xenograft (PDOX) mouse model of a cisplatinum-resistant osteosarcoma lung metastasis that was sensitive to temozolomide and trabectedin: implications for precision oncology

**DOI:** 10.18632/oncotarget.19095

**Published:** 2017-07-08

**Authors:** Kentaro Igarashi, Takashi Murakami, Kei Kawaguchi, Tasuku Kiyuna, Kentaro Miyake, Yong Zhang, Scott D. Nelson, Sarah M. Dry, Yunfeng Li, Jane Yanagawa, Tara A. Russell, Arun S. Singh, Hiroyuki Tsuchiya, Irmina Elliott, Fritz C. Eilber, Robert M. Hoffman

**Affiliations:** ^1^ AntiCancer, Inc., San Diego, California, USA; ^2^ Department of Surgery, University of California, San Diego, California, USA; ^3^ Department of Orthopaedic Surgery, Kanazawa University, Kanazawa, Japan; ^4^ Division of Hematology-Oncology, University of California, Los Angeles, California, USA; ^5^ Department of Pathology, University of California, Los Angeles, California, USA; ^6^ Division of Surgical Oncology, University of California, Los Angeles, California, USA

**Keywords:** osteosarcoma, recurrence, lung metastasis, PDOX, chemotherapy

## Abstract

In the present study, we evaluated the efficacy of trabectedin (TRAB) and temozolomide (TEM) compared to cisplatinum (CDDP) on a patient-derived orthotopic xenogrraft (PDOX) of a lung-metastasis from an osteosarcoma of a patient who failed CDDP therapy. Osteosarcoma resected from the patient was implanted orthotopically in the distal femur of mice to establish PDOX models which were randomized into the following groups when tumor volume reached approximately 100 mm^3^: G1, control without treatment; G2, CDDP (6 mg/kg, intraperitoneal injection, weekly, for 2 weeks); G3, TRAB (0.15 mg/kg, intravenous injection, weekly, for 2 weeks); G4, TEM (25 mg/kg, oral, daily, for 14 days). Tumor size and body weight were measured with calipers and a digital balance, respectively, twice a week. On day 14 after initiation of treatment, TEM and TRAB, but not CDDP, significantly inhibited tumor volume compared to untreated control: control (G1): 814.5±258.8 mm^3^; CDDP (G2): 608.6±126.9 mm^3^; TRAB (G3): 286.6±133.0 mm^3^; TEM (G4): 182.9±69.1 mm^3^. CDDP vs. control, p=0.07; TRAB vs. control, p=0.0004; TEM vs. control p =0.0002; TRAB vs. CDDP, p =0.0002; TEM vs. CDDP, p =0.00003. The results of the present study show that a PDOX model of an osteosarcoma lung-metastasis that recurred after adjuvant CDDP-treatment has identified potentially, highly-effective drugs for this recalcitrant disease, while accurately maintaining the CDDP resistance of the tumor in the patient, thereby demonstrating the potential of the osteosarcoma PDOX model for precision oncology.

## INTRODUCTION

Osteosarcoma is a mesenchymal tumor comprising spindle cells and osteoid formation. Osteosarcoma incidence is greatest in adolescence and again in the seventh and eighth decades. Osteosarcoma first-line therapy is high-dose methotrexate, cisplatinum (CDDP), doxorubicin, and ifosfamide. Metastatic osteosarcoma has a less than 20% long-term survival rate that has not improved for many years [1–7].

Temozolomide (TEM) has been used clinically against high-grade glioma [8], melanoma [9], and pediatric rhabdomyosarcomas [10]. TEM has been tested pre-clinically against osteosarcoma cells combined with a molecular targeting drug [11]. TEM has also been tested as a single-agent against 6 osteosarcoma xenograft models and achieved complete response in two and stable disease in one [12]. Methylation of the O-6-methylguanine-DNA methyltransferase (MGMT) gene is correlated with sensitivity to TEM and may become as a biomarker for TEM. Methylation of the MGMT gene occurred in 23.5% of osteosarcoma patients in one study [13].

Trabectedin (TRAB) is an alkylating agent derived from the Caribbean tunicate, Ecteinascidia turbinate [14]. TRAB has been tested on patients with liposarcoma and leiomyosarcoma [15, 16]. TRAB arrests cells in the G_2_/M phase of the cell cycle [17]. TRAB has shown efficacy against CDDP-resistant bone cancer *in vitro* [18]. TRAB is marketed as Yondelis (Johnson & Johnson, Raritan, NJ) for leiomyosarcoma and liposarcoma. (https://www.cancer.gov/news-events/cancer-currents-blog/2015/fda-trabectedin-sarcoma).

TRAB has been used for recurrent osteosarcoma patients with a 12% partial response rate [19]. Patients with metastatic osteosarcoma that had the wild-type AAP1164 excision-repair cross-complementing 5 (ERCC5) gene had major responses to TRAB [20], suggesting this gene could be a biomarker for TRAB.

We previously reported that a subcutaneous transplant nude-mouse model of an ostoeosarcoma lung metastasis that occurred after adjuvant CDDP treatment of the patient, was regressed by tumor-targeting *Salmonella typhimurium* A1-R (*S. typhimurium* A1-R). The osteosarcoma was only partially sensitive to the molecular-targeting drug sorafenib, which did not arrest its growth. *S. typhimurium* A1-R was significantly more effective than sorafenib [21].

We previously developed the orthotopic intratibial-implantation model using a human osteosarcoma cell line in nude mice [22]. In the intratibial orthotopic osteosarcoma model, we observed by color-coded imaging, when high- and low-metastatic osteosarcoma cell lines were co-transplanted, there was gene exchange between the cell lines *in vivo* resulting in the conversion of the low-metastatic cell line to high-metastatic [23]. In another previous study from our laboratory, the intratibial orthotopic osteosarcoma model used fluorescent-protein-expressing human osteosarcoma cells in order to develop fluorescence-guided surgery (FGS) which reduced multiple lung metastasis and thereby increased mouse survival time [24]. Echistatin, a cyclic RGD peptide, which is an antagonist of αvβ3 integrin (disintegrin), inhibited primary tumor growth as well as pulmonary metastases in a human osteosarcoma cell line in the intratibial orthotopic model used in a previous study in our laboratory [25].

Toward the goal of individualized precision oncology, our laboratory pioneered the patient-derived orthotopic xenograft (PDOX) nude mouse model with the technique of surgical orthotopic implantation (SOI), including pancreatic [26–29], breast [30], ovarian [31], lung [32], cervical [33], colon [34–36], and stomach cancer [37], sarcoma [38–42] and melanoma [43–45].

In the present study, we tested a PDOX model established from an osteosarcoma lung metastasis that recurred after adjuvant cisplatinum treatment of the patient, for sensitivity to CDDP, TRAB and TEM.

## RESULTS AND DISCUSSION

### Efficacy of CDDP, TRAB and TEM on the osteosarcoma PDOX mouse model

**Figure 1:**
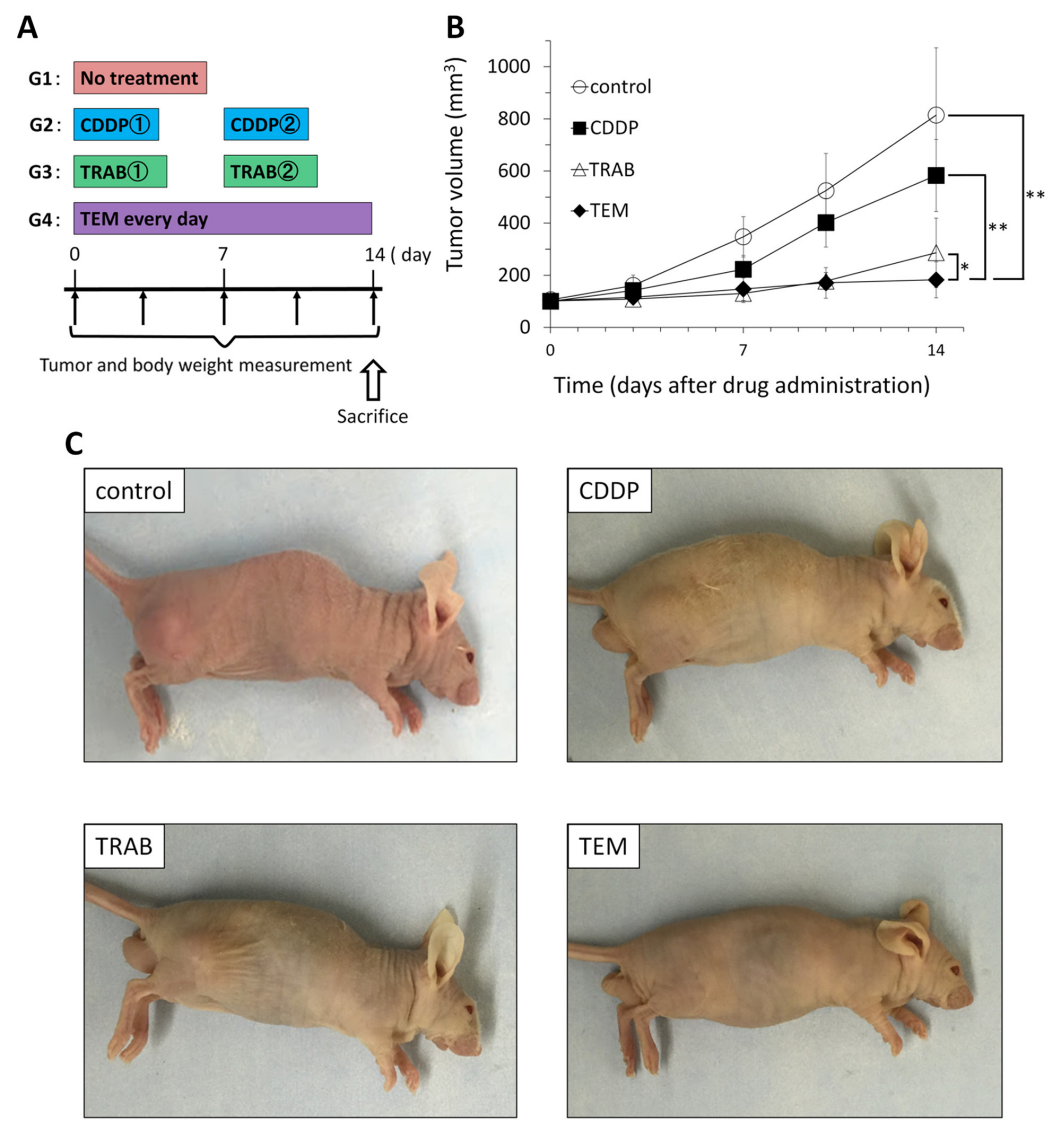
Efficacy of cisplatinum (CDDP), trabectedin (TRAB) and temozolomide (TEM) on the osteosarcoma PDOX Osteosarcoma tissue was grown orthotopically in the right distal femur of nude mice and allowed to form tumors. **(A)** Mice were treated with CDDP (6 mg/kg, i.p., qw×2); TRAB (0.15 mg/kg, i.v., qw×2); and TEM (25 mg/kg, p.o., qd×14). Tumor volume was measured at the indicated time points after the onset of treatment. n=8 mice/group. **(B)** Tumor growth curves of treated and untreated mice. **(C)** Photos of representative treated and untreated osteosarcoma PDOX models. * p< 0.05, **p< 0.001

**Figure 2:**
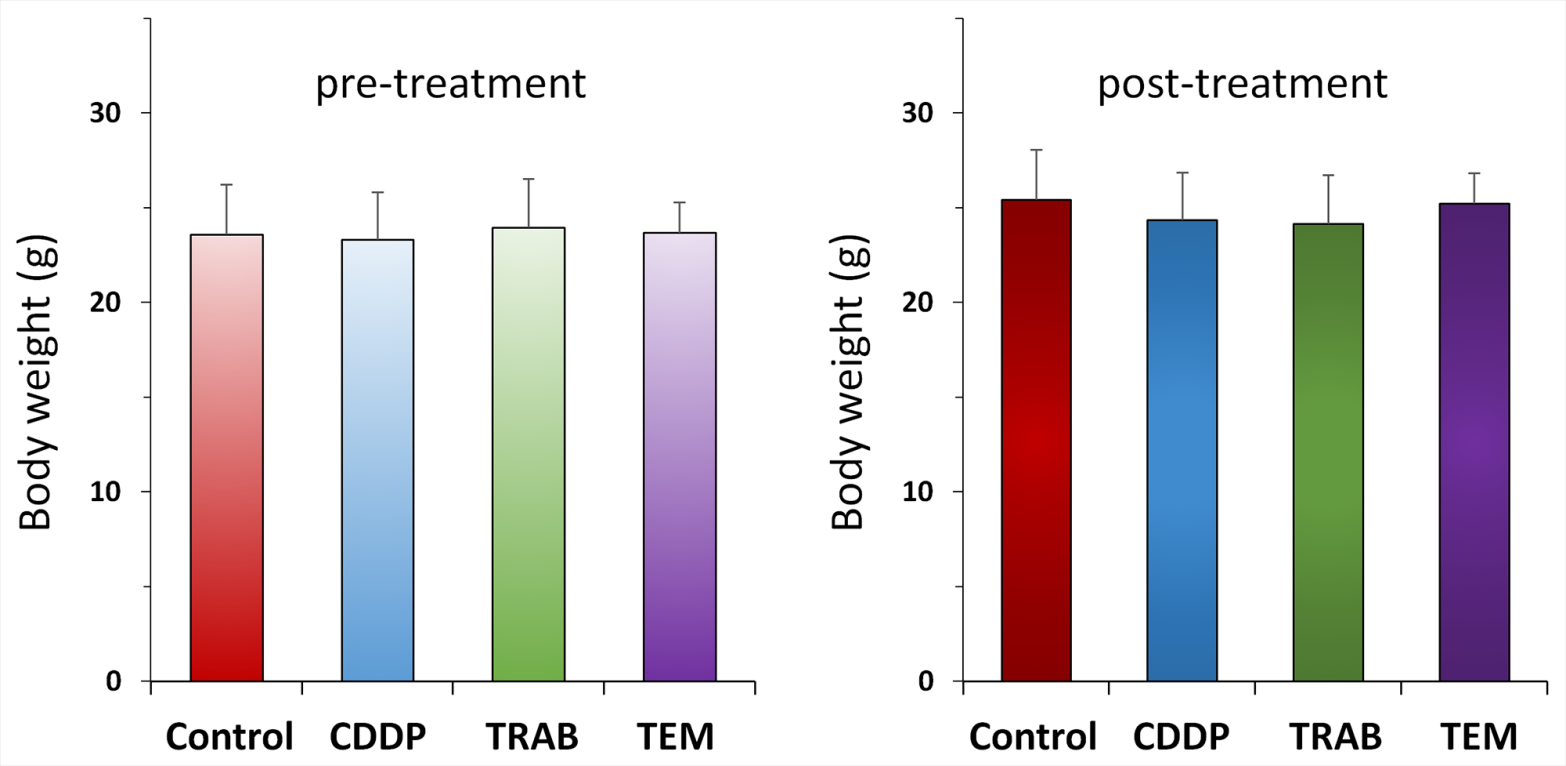
Body weights after CDDP, TRAB and TEM treatment Bar graph shows body weight in each group at pre-treatment and 2 weeks after drug administration

All drugs but CDDP significantly inhibited tumor volume compared to untreated control on day 14 after treatment initiation: control (G1): 814.5±258.8 mm^3^; CDDP (G2): 608.6±126.9 mm^3^; TRAB (G3): 286.6±133.0 mm^3^; TEM (G4): 182.9±69.1 mm^3^. CDDP vs. control, *p*=0.07; TRAB vs. control, *p*=0.0004; TEM vs. control, *p* =0.0002; TRAB vs. CDDP, *p* =0.0002; TEM vs. CDDP, *p* =0.00003; TEM vs. TRAB, p=0.025 (Figure 1). There were no animal deaths in any group. Body weight of the treated mice was not significantly different from untreated control in any group (Figure 2).

### Histology of original patient tumor and control and treated tumors

**Figure 3:**
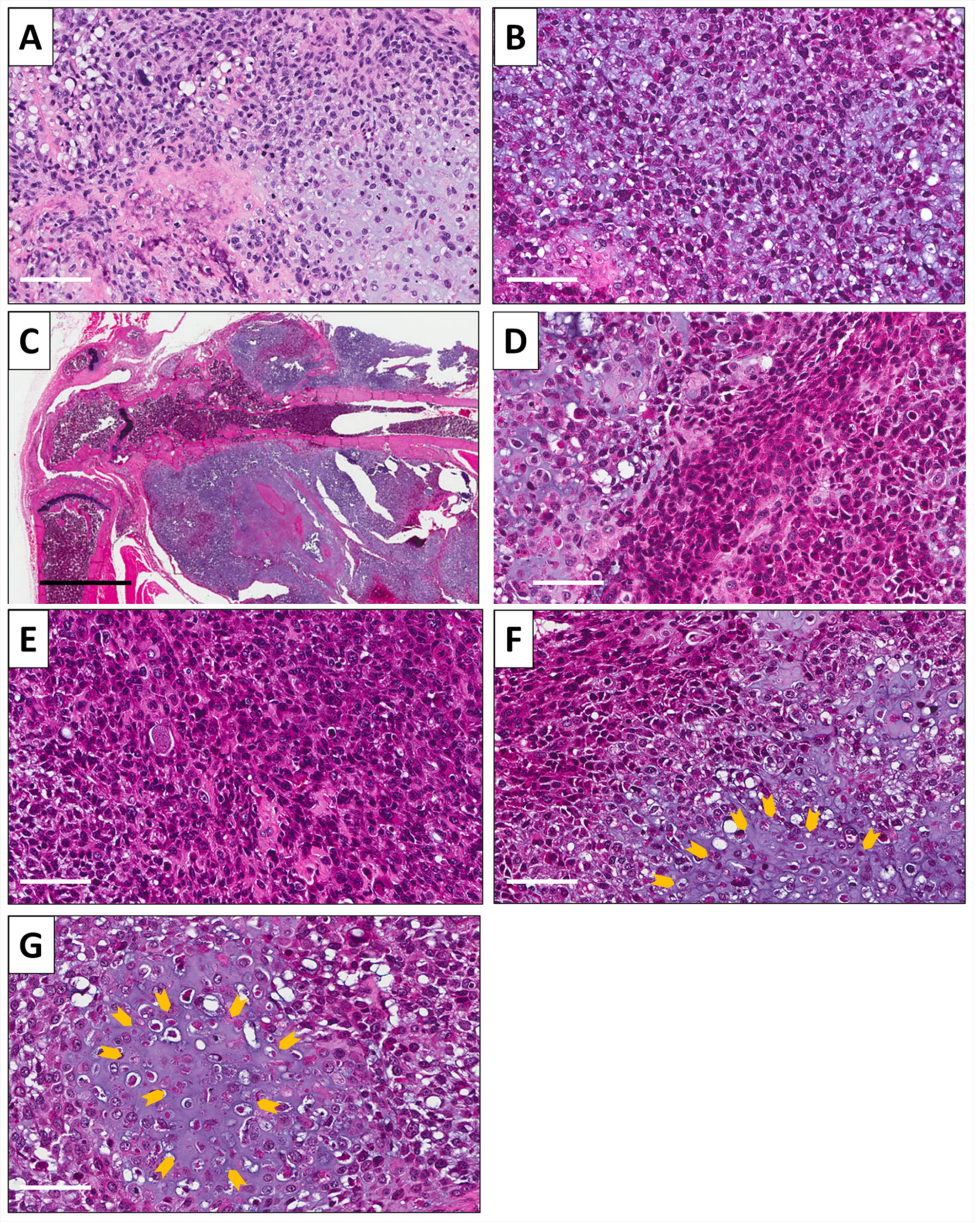
Osteosarcoma histology H&E-stained sections of **(A)** the original patient tumor (micro). (**B)** Subcutaneously-implanted tumor (micro). **(C)** Orthotopically-implanted tumor (macro). **(D)** Orthotopically-implanted tumor (micro). **(E)** PDOX tumor treated with CDDP. **(F)** PDOX tumor treated with TRAB. **(G)** PDOX tumor treated with TEM. Necrotic areas are indicated by yellow arrows. Scale bars: 100 μm for (A, B, D). Black scale bar: 2 mm for C. Scale bars: 80 μm for (E-G).

Hematoxylin and eosin (H&E)-stained sections were made from the patient and mouse-grown tumors. The original patient osteosarcoma tumor had hypercellular areas populated by anaplastic cells displaying nuclear pleomorphism, coarse and hyperchromatic chromatin and abundant mitotic figures (Figure 3A). The subcutaneously-implanted mouse-grown tumor contained hypercellular areas with anaplastic cells displaying nuclear pleomorphism, coarse and hyperchromatic chromatin and mitotic figures. A chondroid matrix was less abundant compared to the original patient tumor (Figure 3B). The low magnification image of the untreated PDOX tumor shows cortical and medullary infiltration of tumor as well as muscle infiltration (Figure 3C). A high power photomicrograph of the untreated PDOX tumor shows a chondroblastic appearance similar to the patient’s original tumor with hypercellular areas filled with tumor cells displaying nuclear pleomorphism and mitotic figures (Figure 3D). PDOX tumors treated with CDDP were comprised of viable cells without apparent necrosis or inflammatory changes and almost the same features compared to the untreated control (Figure 3E). Tumors treated with TRAB showed changes in cancer-cell shape with slight necrosis (Figure 3F). TEM-treated tumors showed more extensive tumor necrosis (Figure 3G), which is consistent with the superior efficacy of TEM.

Recurrent metastatic osteosarcoma that has failed first-line therapy is a recalcitrant cancer. The results of the present study suggest that novel active drugs can be identified for this disease using the PDOX model. Further studies are necessary to determine the clinical significant of the present findings. The PDOX model of lung metastatic osteosarcoma that failed CDDP treatment replicated the clinical outcome of CDDP treatment of the patient.

Previously-developed concepts and strategies of highly-selective tumor targeting can take advantage of molecular targeting of tumors, including tissue-selective therapy which focuses on unique differences between normal and tumor tissues [46–51].

## MATERIALS AND METHODS

### Mice

Athymic nu/nu nude mice (AntiCancer Inc., San Diego, CA), 4–6 weeks old, were used in this study. Animals were housed in a barrier facility on a high efficiency particulate arrestance (HEPA)-filtered rack under standard conditions of 12-hour light/dark cycles. The animals were fed an autoclaved laboratory rodent diet. All animal studies were conducted with an AntiCancer Institutional Animal Care and Use Committee (IACUC)-protocol specifically approved for this study and in accordance with the principles and procedures outlined in the National Institute of Health Guide for the Care and Use of Animals under Assurance Number A3873-1. In order to minimize any suffering of the animals, anesthesia and analgesics were used for all surgical experiments. Animals were anesthetized by subcutaneous injection of a 0.02 ml solution of 20 mg/kg ketamine, 15.2 mg/kg xylazine, and 0.48 mg/kg acepromazine maleate. The response of animals during surgery was monitored to ensure adequate depth of anesthesia. The animals were observed on a daily basis and humanely sacrificed by CO_2_ inhalation when they met the following humane endpoint criteria: severe tumor burden (more than 20 mm in diameter), prostration, significant body weight loss, difficulty breathing, rotational motion and body temperature drop.

### Patient-derived tumor

The study was previously reviewed and approved by the UCLA Institutional Review Board (IRB #10-001857) [17]. Written informed consent was obtained from the patient as part of the above-mentioned UCLA Institutional Review Board-approved protocol. A 16-year old patient with localized left-distal-femoral high-grade osteosarcoma underwent CDDP-based neo-adjuvant chemotherapy and limb salvage with distal femoral replacement. One year later, the osteosarcoma recurred with three bilateral metachronous pulmonary metastases. The patient was treated with curative surgery at the Division of Surgical Oncology, University of California, Los Angeles (UCLA). The patient did not receive chemotherapy or radiotherapy after tumor occurrence prior to lung surgery [21].

### Surgical orthotopic implantation (SOI) for establishment of the PDOX osteosarcoma model

**Figure 4:**
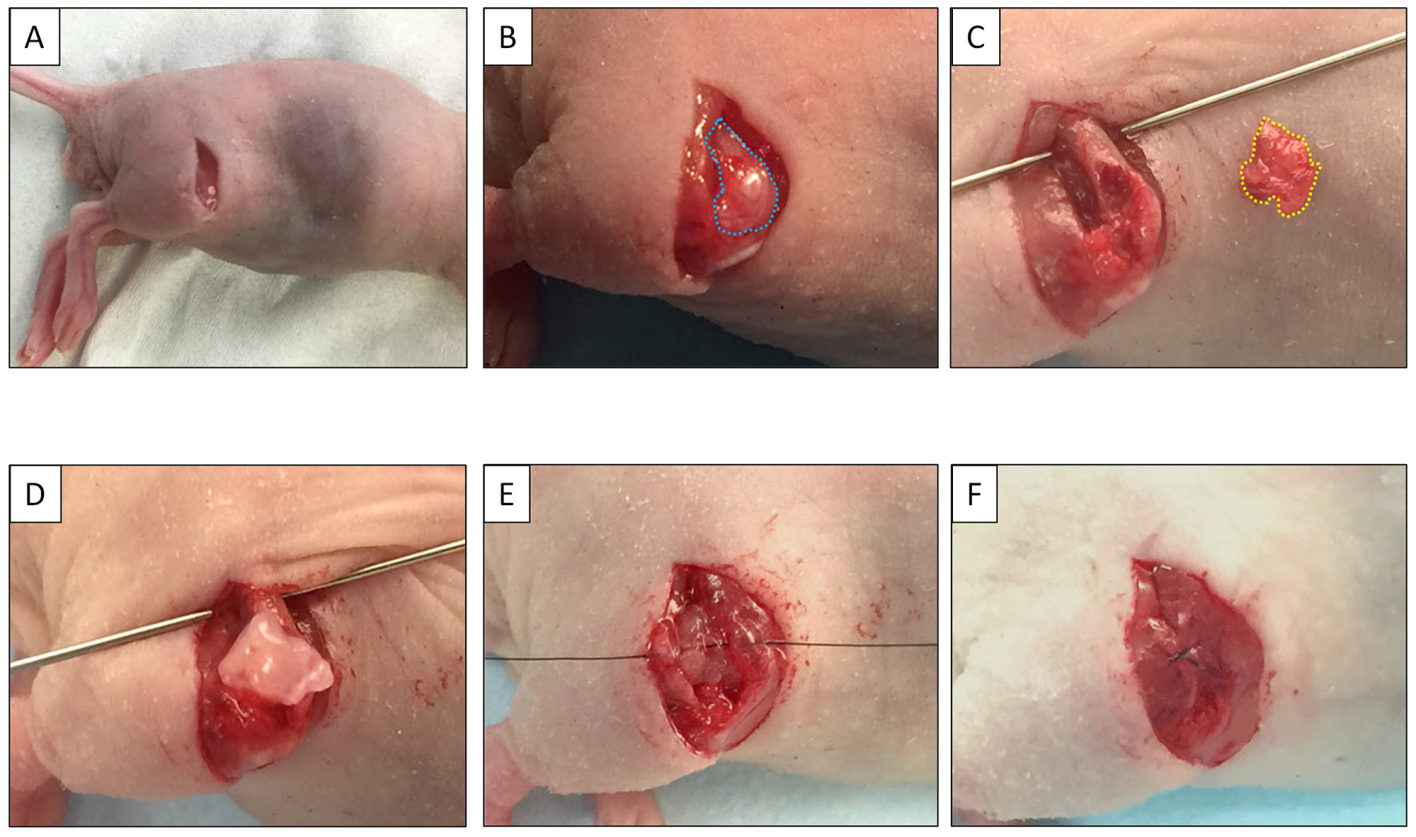
Establishment of the osteosarcoma PDOX model **(A)** A skin incision was made on the right thigh. **(B)** The vastas lateralis muscle and biceps femoris muscle were split to reach the distal femur. **(C)** The lateral condyle of the distal femur was resected. **(D)** A single 3 mm tumor fragment was implanted orthotopically into the space. **(E)** The muscle was closed with a 6-0 nylon suture. **(F)** The tumor fragment was totally covered by the muscle.

A lung metastasis from the osteosarcoma patient was previously established subcutaneously in mice [21]. Subcutaneously grown tumors were harvested and cut into small fragments (3-4 mm). After nude mice were anesthetized, a 10 mm skin incision was made on the right thigh, the vastus lateralis muscle was opened and the biceps femoris muscle was split to reach the distal femur. An incision was made in the lateral patello-femoral ligament, sparing the knee joint and then the lateral condyle of the femur was resected. A single 3 to 4 mm tumor fragment was implanted orthotopically into the space to establish a PDOX model. The muscle and wound was closed with 6-0 nylon suture (Ethilon, Ethicon, Inc., NJ, USA) (Figure 4).

### Treatment design

The PDOX models were randomized into the following groups when tumor volumes reached 100 mm^3^: G1, control without treatment, n=8; G2, CDDP (6 mg/kg, intraperitoneal [i.p.] qw×2, n=8); G3, TRAB (0.15 mg/kg, intravenous [i.v.], qw×2, n=8); G4, TEM (25 mg/kg, per oral [p.o.], qd×14, n=8). The schedule for TRAB and TEM, as well as CDDP, were obtained from previously published results [43–45, 52]. Tumor length, width and mouse body weight were measured twice in a week. Tumor volume was calculated by following formula: Tumor volume (mm^3^) = length (mm) × width (mm) × width (mm) × 1/2. Data are presented as mean ± SD.

### Histological analysis

Fresh tumor samples were fixed in 10% formalin and embedded in paraffin before sectioning and staining. Tissue sections (3 μm) were deparaffinized in xylene and rehydrated in an ethanol series. Hematoxylin and eosin (H&E) staining was performed according to standard protocol. Histological examination was performed with a BHS system microscope. Images were acquired with INFINITY ANALYZE software (Lumenera Corporation, Ottawa, Canada).

## CONCLUSIONS

In the present study, the PDOX model identified TEM and TRAB as potential effective drugs for a recurrent, recalcitrant, CDDP-resistant metastatic osteosarcoma. The PDOX model precisely replicated the CDDP resistance of the patients recurring lung metastasis, suggesting the patient will have a possibility to respond to TEM or TRAB, which were effective in the PDOX model. Our results are of particular importance for TRAB, which is marketed as Yondelis for leiomyosarcoma and liposarcoma, since the results demonstrate activity of this drug in CDDP-resistant metastatic osteosarcoma. However, further studies are needed to determine the clinical potential of TRAB for this disease. In particular, more effective schedules of TRAB, as well as combination drugs need to be identified in the PDOX models at doses that can also be achieved clinically.

Future experiments will include mechanistic experiments on the differential activity of TRAB and TEM compared to CDDP as well as identifying molecular-signaling pathways that are activated by these compounds, especially in PDOX models of patients that express biomarkers for these drugs.

Our results show the potential of the PDOX model for refractory, recurrent osteosarcoma model for precision oncology.

Both TRAB and TEM showed strong inhibition (Figure 1B, 1C) almost arresting tumor growth and causing extensive tumor necrosis (Figure 3F, 3G). This very promising result will be used in future experiments to design combination drug experiments in order to obtain tumor regression in PDOX models and thereby be of potential use for clinical efficacy.
